# Benchmarking Sternotomy in Isolated Left Anterior Descending Disease: The Need to Account for the Full Selection Pathway

**DOI:** 10.1093/icvts/ivag124

**Published:** 2026-04-21

**Authors:** Fatih Kizilyel, Bedirhan Bugra Bayici

**Affiliations:** Department of Cardiovascular Surgery, Kosuyolu High Specialization Education and Research Hospital, Istanbul, Turkey; Department of Cardiovascular Surgery, Kosuyolu High Specialization Education and Research Hospital, Istanbul, Turkey

Dear Editor,

We read with great interest the national study by Sampon et al. evaluating median sternotomy coronary artery bypass grafting for isolated left anterior descending (LAD) artery disease.^1^ The authors should be commended for presenting a substantial contemporary cohort and demonstrating excellent overall outcomes, with low 30-day and 1-year mortality and no significant survival difference between off-pump (OPCAB) and on-pump (ONCAB) strategies after propensity matching. Their effort to position sternotomy-based LAD revascularization as a benchmark dataset for future minimally invasive studies is timely and clinically relevant.

We believe, however, that the interpretation of the OPCAB–ONCAB comparison requires caution because the factors guiding treatment selection are insufficiently characterized. In routine practice, the choice of revascularization strategy is shaped by detailed anatomical and clinical considerations assessed by the Heart Team.^2^ Yet this analysis is confined to median sternotomy cases and therefore does not capture the broader rationale for choosing sternotomy rather than PCI or a minimally invasive thoracotomy-based approach in these patients.

This issue is particularly relevant because, after matching, the most notable difference was target vessel reintervention (TVR), which favored ONCAB. The authors appropriately acknowledge important registry limitations, including the absence of surgeon-level experience and uncertainty regarding the distinction between true single-vessel and multivessel disease. However, these unmeasured factors remain central to interpretation. The observed difference in TVR may therefore reflect residual confounding related to anatomical complexity or completeness of revascularization, rather than pump strategy alone.

We also believe that the concept of a “benchmark” should be framed cautiously. Given that minimally invasive procedures accounted for an increasing proportion of surgical volume over time, a sternotomy-only dataset represents an important historical and procedural reference, but not necessarily a complete contemporary benchmark for isolated LAD disease. In current practice, the relevant comparison is often not simply OPCAB versus ONCAB, but sternotomy-based CABG versus minimally invasive direct coronary artery bypass (MIDCAB) and PCI.^3^ Recent literature supports MIDCAB as an increasingly relevant comparator in isolated LAD revascularization.^4^

Future comparative studies incorporating anatomical suitability, completeness of revascularization, and minimally invasive cohorts would therefore be highly valuable. We would be interested to know whether the authors plan to analyze the excluded minimally invasive group within this registry to provide a more comprehensive picture of contemporary LAD revascularization outcomes.

## Data Availability

The data underlying this article will be shared on reasonable request to the corresponding author.
